# DCSO: towards an ontology for machine-actionable data management plans

**DOI:** 10.1186/s13326-022-00274-4

**Published:** 2022-07-26

**Authors:** João Cardoso, Leyla J. Castro, Fajar J. Ekaputra, Marie C. Jacquemot, Marek Suchánek, Tomasz Miksa, José Borbinha

**Affiliations:** 1 INESC-ID, R. Alves Redol, 9, Lisboa, 1000-029 Portugal; 2 Instituto Superior Técnico, Universidade de Lisboa, Av. Rovisco Pais, 1, Lisboa, 1049-001 Portugal; 3 ZB MED Information Centre for Life Sciences, Gleueler Straße, 60, Cologne, 50931 Germany; 4 TU Wien, Karlsplatz, 13, Vienna, 1040 Austria; 5 DMP OPIDoR, R. Jean Zay, 2, Vandoeuvre-lès-Nancy, 54519 France; 6 Inist-CNRS, R. Jean Zay, 2, Vandoeuvre-lès-Nancy, 54500 France; 7 Faculty of Information Technology, Czech Technical University in Prague, Thákurova, 9, Prague, 160 00 Czechia; 8 SBA Research, Floragasse, 7, Vienna, 1040 Austria

**Keywords:** Data management plan, Machine-actionable data management plan, Ontology, Semantic web technologies

## Abstract

The concept of Data Management Plan (DMP) has emerged as a fundamental tool to help researchers through the systematical management of data. The Research Data Alliance DMP Common Standard (DCS) working group developed a set of universal concepts characterising a DMP so it can be represented as a machine-actionable artefact, i.e., machine-actionable Data Management Plan (maDMP). The technology-agnostic approach of the current maDMP specification: (i) does not explicitly link to related data models or ontologies, (ii) has no standardised way to describe controlled vocabularies, and (iii) is extensible but has no clear mechanism to distinguish between the core specification and its extensions.This paper reports on a community effort to create the DMP Common Standard Ontology (DCSO) as a serialisation of the DCS core concepts, with a particular focus on a detailed description of the components of the ontology. Our initial result shows that the proposed DCSO can become a suitable candidate for a reference serialisation of the DMP Common Standard.

## Background

With the continuous growth of research data and the ultimate goal of sharing FAIR (Findable, Accessible, Interoperable, and Reusable) data [1], researchers face the challenge of systematically managing that data and its corresponding metadata. Data Management Plans (DMPs) make it easier for researchers to respond to this challenge. The DMP, produced as a text-based document, describes techniques, methods and policies on how data is produced and managed throughout its life cycle [2]. Additionally, it establishes associations between data management activities and the corresponding responsible actors [3].

The concept of DMP as a document has evolved towards a machine-actionable DMP (maDMP). The implementation of maDMPs will allow to overcome some obstacles related to the current text-based representation [4]. One of the main issues is the level of details provided by a DMP, which can vary according to the design choices, awareness, and knowledge of its creators. In addition, having information expressed as free-text often leads to DMPs with incomplete, inadequate, ambiguous or missing information. The main goal of maDMPs is representing DMPs with a format that makes its information readable and reusable by both humans and automated systems. This machine-actionable representation will allow the exchange of information between automated systems, its integration into existing data management workflows, and will support automated machine-process pipelines regarding data management policies [5, 6]. As a consequence, it will lessen the administrative burden for researchers and facilitate the involvement and support from data management experts and services. Furthermore, it will ease the updating process of DMPs and data management follow-ups, and enable linking of research outputs, research objects, actors, and infrastructures. As a result, DMPs, being machine-actionable, will facilitate the actual implementation of the FAIR principles.

As machine-actionable representations require certain formalism and standardisation levels, the Research Data Alliance (RDA)^1^ DMP Common Standards (DCS) working group created a universal characterisation for DMPs formalised as the DCS application profile. It also released its corresponding serialisation using the JavaScript Object Notation (JSON) format^2^, leaving the responsibility of developing other serialisation formats to the community. Although the DCS application profile is a step further regarding text-based DMPs, there are still a number of open challenges related to interoperability, in particular (1) the lack of explicit linking to existing ontologies; (2) the lack of mechanisms to describe controlled vocabularies; and (3) the lack of a mechanism that allowed for the extension of DCS set of terms. In order to tackle these challenges and boost interoperability, there is a need for a semantic layer added on top of the syntactic one provided by the DCS application profile.

This paper reports on the creation of the DMP Common Standard ontology (DCSO), currently on its 4.0.0 version, a community effort towards the creation of an ontology serialisation of the DCS application profile. The creation of DCSO aimed to address the aforementioned challenges with the current DCS model and existing serialisations.

The remaining of this paper details the resulting ontology and its components, and is organised as follows. “Construction and content” section reports on the creation process of DCSO. The narrative that is presented starts with the motivation behind the creation of the DCSO, and follows the challenges faced in its multiple versions up to its latest (version 4.0.0). All of the reported work is framed within a wider scope of having the DCSO adopted as the official semantic-based serialisation of the DCS application profile. “Utility and discussion” section presents a use case, through the description of the adoption of DCSO by the Data Stewardship Wizard (DSW) DMP creation tool [7]. Finally, “Conclusions” section provides a summarised review on the contents of this paper, as well as a description of the future goals for DCSO.

## Construction and content

This section will describe the DCSO creation process, as well as the methodology used to iteratively develop the DCSO. “The application profile as starting point” section offers a brief characterisation of the DCS application profile and describes the key requirements to be accomplished by the DCSO. “Initial version” section follows with a description of the DCSO origins by focusing on its initial version (2.0.2). The first stable version (3.0.2) is subsequently described in “First stable version” section. This version was a complete redesign of the DCSO, in an effort that took place during the RDA Hackathon on Machine-Actionable Data Management Plans in 2020 [8]. And finally, the latest (4.0.0) version of the DCSO is characterised in “Towards DCSO adoption by the DCS working group” section. It primarily reports on a number of pre-requisites which the first stable version did not meet, and needed to be addressed by the creation of a new version. The compliance with these pre-requisites and the resulting DCSO version 4.0.0 is part of an ongoing effort to have the DCSO adopted by the DCS working group as the official serialisation of the DCS application profile.

### The application profile as starting point

To comply with the goal of establishing a set of universal terms to characterise a DMP, the DCS working group has strived to create an application profile. According to the definition, an application profile is a metadata design specification that uses a selection of terms from multiple metadata vocabularies, with added constraints, to meet application-specific requirements^3^. However, due to the fact that not all of the terms selected by the DCS working group are yet associated with established metadata vocabularies, the concept of application profile is not fully materialised, and therefore it is still an ongoing task. Regardless of this fact, and as part of the overall goal, there was the need to develop serialisations of the application profile. These would allow any tools or systems engaged in research data processing, not only to consume data but also to add data to maDMPs, thus automating data interchange.

The DCSO was created as a community initiative with the overall goal of expanding the set of existing serialisations of the DCS application profile, by adding a semantic-based serialisation. With DCSO, information from the DCS application profile is represented using semantic technologies, specifically ontologies, which allow for the representation of a shared conceptualisation of knowledge through the usage of formal semantics [9]. One of the key characteristics behind the selection of ontologies was their extensibility, as concepts can be matched or relations established between ontologies covering distinct domains. This characteristic enforces the suitability of ontologies as a means to represent the DCS application profile, for it is also designed with modularity in mind. Additionally, ontologies enable reasoning, and thereby knowledge inference from the information explicitly represented [10]. In spite of the traditional perception of ontologies as highly formal means of knowledge representation, their suitability for creation of Linked Open Data (LOD) has been proven [11, 12]. The usage of semantic technologies is therefore in compliance with the overarching requirements established by the concept of a maDMP.

In the process of creating a semantic-based serialisation of the DCS application profile, three key requirements that DCSO should accomplish were identified. These are: (1) DCSO should allow for ontologies referenced in the DCS application profile to be integrated through the reuse of its terms; (2) DCSO should allow and enforce the usage of controlled vocabularies; and (3) DCSO should be extendable, so as to comply with any future extensions of the DCS application profile. The following sections describe DCSO creation process, from its origins to its current iteration.

### Initial version

The first attempt at creating a semantic-based serialisation of the DCS application profile took place in the spring of 2019. This version (2.0.2) was created to serve primarily as a proof of concept. Thus, the creation process was expedited and simplified by not fully abiding by the best practices in ontology engineering. This first version proved the viability of a semantic-based representation of the DCS application profile, however, it also failed in fully complying with the three key requirements that the DCSO concept should accomplish.

The DCS application profile references multiple terms and fields from standardised vocabularies (e.g., Data Catalog Vocabulary (DCAT)^4^ and Dublin Core (DC)^5^). In this initial version of DCSO, all of the terms and fields were redefined. This was contrary to the best practice of reusing terms through the integration of existing domain ontologies. In spite of having all terms and fields represented, the lack of reuse of referenced terms and fields meant that this version of DCSO would not meet one of the key requirements for its development.

In order to simplify the creation process, it was decided that a set of custom literal datatypes was to be created to accommodate the usage of the controlled vocabularies specified in the DCS application profile. This solution is in breach of the best practices of ontology engineering, and albeit users of DCSO could potentially use controlled vocabularies, this was not a scalable solution moving forward. In addition to this, the decision was made to represent multiplicity and type constraints through the usage of Web Ontology Language (OWL) constraints. Despite this being a viable means of constraint representation in ontology engineering, this solution would not allow for compliance validation of the data with the specification. As a result it was, for all effective purposes, impossible to enforce the use of controlled vocabularies, thus failing to comply with yet another key requirement.

Finally, there were also other issues in the pursuit of following the best practices in ontology engineering, such as using the digital repository links as the ontology namespace, opposed to assigning a persistent namespace via a Uniform Resource Identifier (URI).

### First stable version

After this initial attempt, the community decided to develop a stable version that comply with the three key requirements for DCSO and follow the best practices in ontology engineering. The RDA Hackathon on Machine-Actionable Data Management Plans [8] proved to be the perfect opportunity to tackle this objective. The motivation for the hackathon was to promote the usage of the maDMP concept by the research community. Participants were encouraged to submit topics and assemble teams that would collaborate for two days to tackle the submitted topics. In line with the motivation of the hackathon, it was decided that the creation of a stable version of DCSO should be one of the proposed topics in the hackathon.

**Fig. 1:**
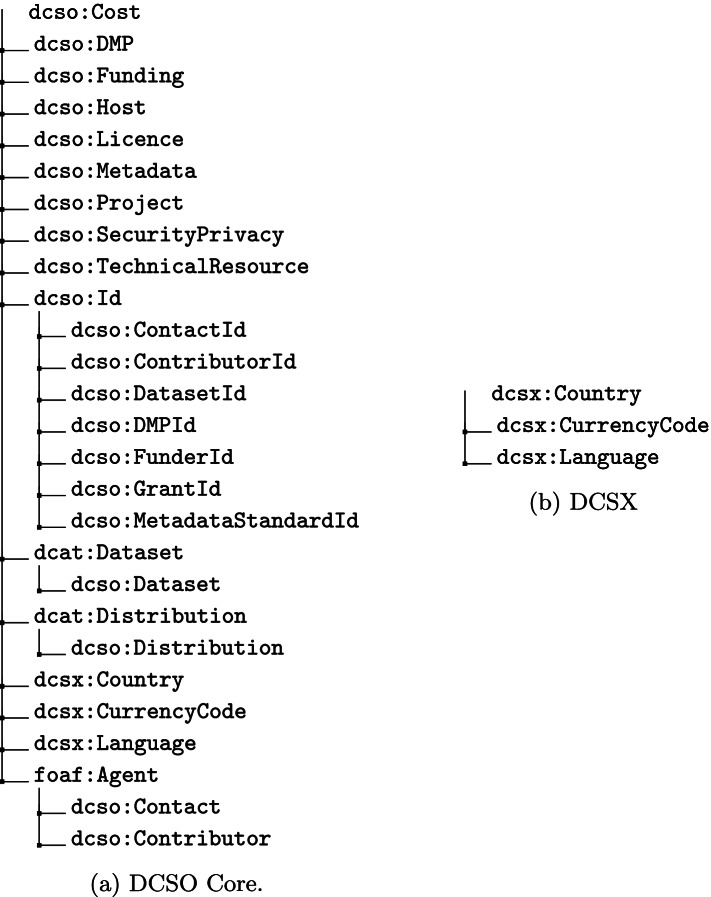
The class structure of DCSO. **a** The class structure of the DCSO Core. **b** The class structure of the DCSX

The new version of DCSO was to be a fresh start, that would benefit from the experience acquired during the creation of the first version. As such, it should comply with all of the previously identified key requirements, whilst following the best practices in ontology engineering. In order to achieve this objective, it was decided that DCSO was to be organised into DCSO Core and DCSO Extensions (DCSX) (as can be seen in Fig. 1). The first would be a representation of the DCS application profile, which would reuse terms from a selection of domain ontologies, whilst the later would support DCSO core by providing an aggregation of all the controlled vocabularies referenced in the DCS application profile. Due to the time constraints imposed by the duration of the hackathon the development process was divided into three iterative stages, resulting in the first stable DCSO version (3.0.2).

#### First stage - DCSO core

The first stage focused solely on the development of DCSO core. The resulting ontology was serialised using the Terse Resource Description Framework (RDF) Triple syntax (Turtle)^6^. In DCSO Core all relations between DCS application profile concepts are represented as object properties, whilst data properties are used to represent DCS application profile terms, as can be seen in Fig. 1a. The object properties are named after the class to which they pertain, using a CamelCase notation, and using the prefix ’has’. This solution solved an existing issue with the DCS application profile, where relations between concepts were left unnamed, with only information regarding their cardinality being provided. The data properties followed a similar naming convention, with the distinction being that no prefix was added. However, some of the terms in the DCS application profile required compliance with controlled vocabularies. The solution to represent this select set of terms was to use object properties establishing a relation between classes of DCSO Core and classes of DCSX (see “Second stage - DCSX and validation layer” section). These object properties followed the same naming convention as those representing relations between concepts in the DCS application profile, e.g., dcso:hasCurrencyCode.

#### Second stage - DCSX and validation layer

The second stage had two objectives: (1) The incorporation of controlled vocabularies in DCSO; and (2) the creation of a constraint validation layer. The DCS application profile specifies a set of three controlled vocabularies: (1) International Organization for Standardization (ISO) 639-3 [13], whose language codes are used to represent the language in which multiple concepts (e.g., *dataset*, *distribution*, and *metadata*) are expressed; (2) ISO 3166-1, whose country codes are used to describe the geographical location of where data is hosted; and (3) ISO 4217 [14], whose currency codes are used to identify the currency in which costs associated with data management are being described in the DMP.

In the initial version of DCSO controlled vocabularies were represented using custom literal datatypes. This solution, implemented solely as a means of simplifying the creation process, was inadequate for a stable version. The hackathon team opted to resort to the creation of a separate ontology that would serve as an extension to DCSO, and where controlled vocabularies could be represented by classes and their terms as individual instances of those classes. The result was the creation of DCSX, and its classes (*dcsx:Language*, *dcsx:Country* and *dcsx:CurrencyCode*), each associated with a set of individual instances, as can be seen in Fig. 1b. Additionally, the necessary object properties required to establish relations to classes belonging to DCSO Core were also created.

The motivation for the creation of DCSO constraints validation layer was to provide users with the means to assess the compliance of their maDMPs with the DCS application profile, whilst also promoting data completeness and consistency. In the initial version of DCSO, constraints were represented using the OWL language; however, due to the limitations of OWL, it was impossible to validate the compliance of individual DMP instances with DCSO. A solution to this issue was to select a constraint representation language that allowed for compliance validation.

Three validation languages are generally regarded as most prevalent: (1) JSON schema; (2) Shape Expression (ShEx) [15]; and (3) the Shapes Constraint Language (SHACL) [16]. None of the three validation languages stands out in terms of dedicated usage in semantic data validation scenarios. We chose ShEx as this is the option used to validate schemas in Wikidata, providing already a big community of practice. Wikidata^7^ is an open knowledge base corresponding to structured data for Wikimedia sister projects, e.g., Wikepedia. In Wikidata, ShEx shapes are supported by the WikiProject Schemas^8^, which includes access to examples and tutorials. ShEx is a data modelling language used to describe RDF graphs. Sets of individual ShEx expressions are collected into a ShEx schema, defining conditions on element relations, their cardinality (e.g., one or more, zero or more, zero or one, etc.) and their existence (e.g., mandatory or optional).

**Fig. 2:**
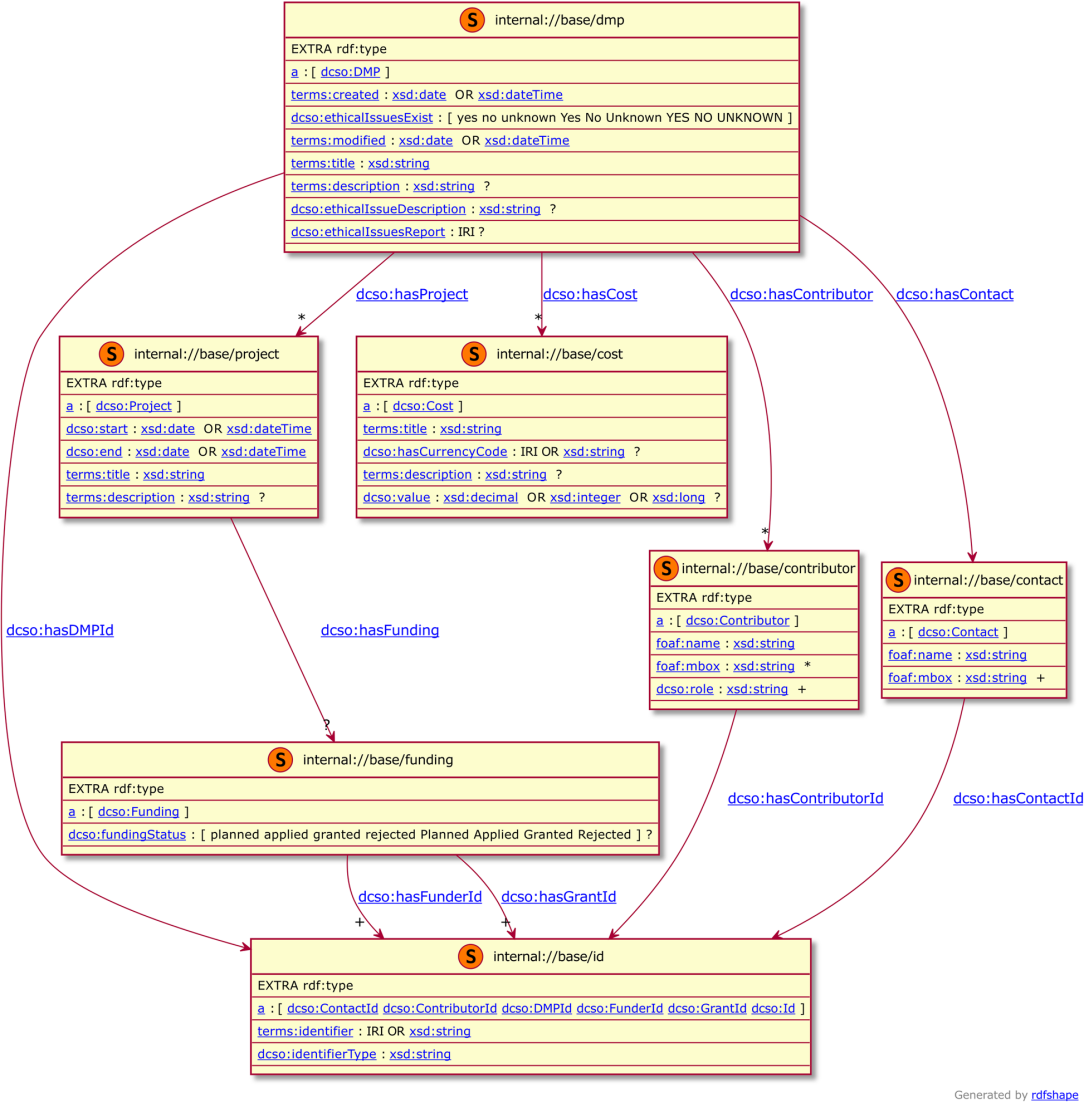
Diagram showing the shapes describing a DMP according to DCSO together with additional elements describing the associated project, cost, contributors and contacts. Validation related to Datasets is not included in this diagram

**Fig. 3:**
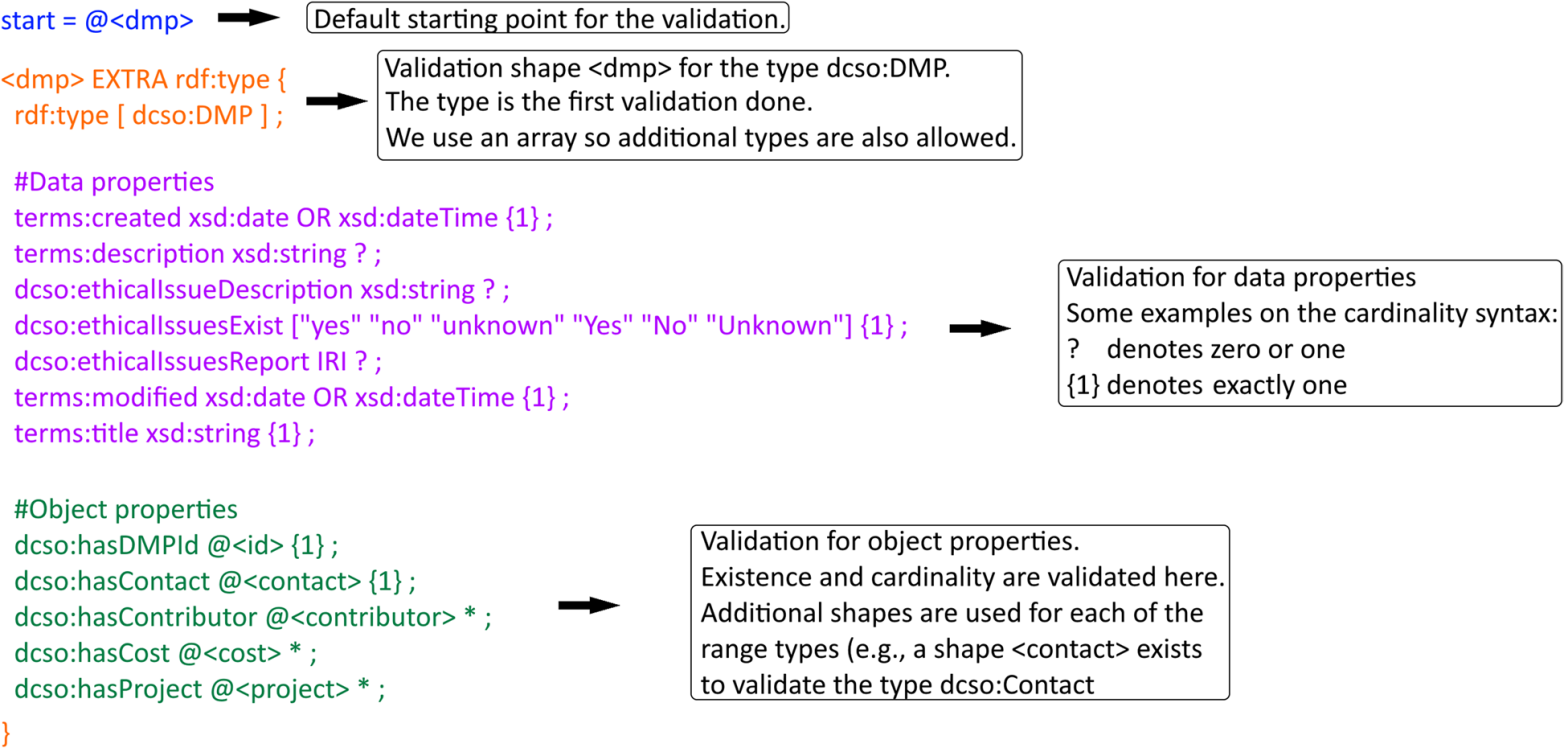
Excerpt of a ShEx schema validating a DMP

DCSO constraint validation layer comprises two distinct ShEx schemas, that follow the constraints established in the DCS application profile. The first schema named ’dcso-dmp’, focuses on the validation of the DMP document. As such, it comprises of elements targeting identifiers, contacts, contributors, costs and projects. The second schema named ’dcso-dataset’, focuses solely on the validation of the datasets referenced in the DMP document. This modularity improves readability (for humans) and makes extensions easier to create. Within a schema, one shape per DCSO class is provided with and initial validation of data properties (e.g., dates or strings) and then a validation of relations expressed by object properties. Validation elements related to the dcso:dmp class are shown in Fig. 2. An explained excerpt corresponding to these shapes is shown in Fig. 3.

**Fig. 4:**
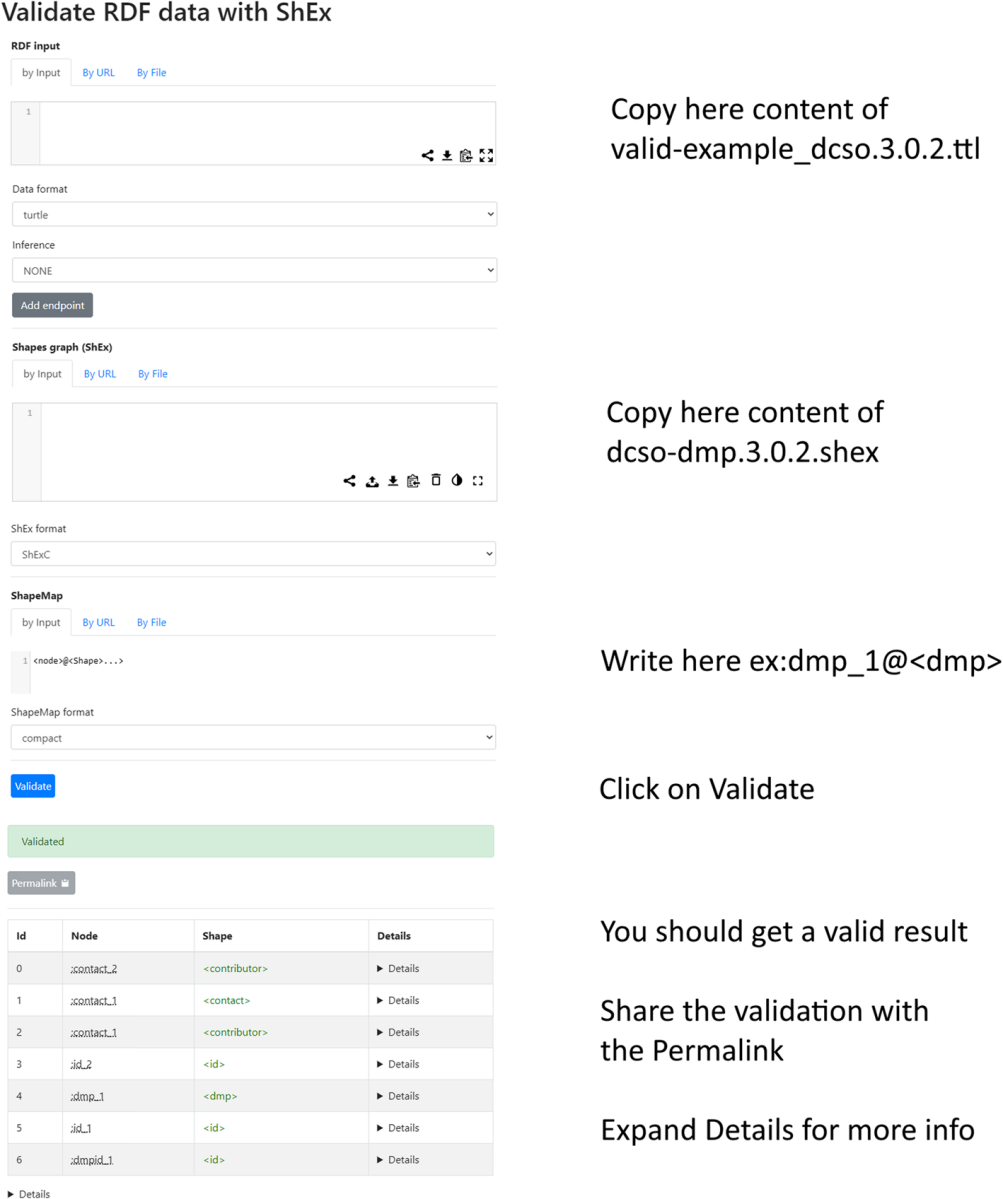
Example of a validation using RDFShape validation service

There are different third-party tools to try and test ShEx schemas, for instance the RDfShape validation service supported by the Web Semantics Oviedo Research Group [17, 18] and ShEx2 Simple Online Validator^9^ supported by ShEx Shape Expressions^10^. Both of them provide an end-user interface where users can directly enter an instance to be validated as well as the corresponding ShEx schema. RDFShape supports direct input, by URL or by file while the ShEx2 Simple Online Validator only supports direct input. In Tabel 1, we describe the process step by step on how to use both the RDFShape and the ShEx2 Simple Online Validator to validate a DMP expressed using the DCSO (this process is also illustrated in Fig 4).

**Table 1:** Steps for Validation DCSO instances using ShEx

RDFShape	ShEx2 Simple Online Validator
1. Go to DCSO validation directory in GitHub^a^;	1. Go to DCSO validation directory^b^ in GitHub;
2. On a different browser window or tab, go to the RDFShape validator service^c^;	2. On a different browser window or tab, go to the ShEx.io validator service;
3. On Github, copy the content of a valid DMP example from the file *valid-dmp-example_dcso.3.0.2.ttl*;	3. On Github, copy the content of a valid DMP example from the file *valid-dmp-example_dcso.3.0.2.ttl*;
4. Paste that valid example on the first text area of the RDFShape validation service, (e.g., RDF input);	4. Paste that valid example on the right text area of ShEx2 Validator;
5. On GitHub, copy the content of the DCSO ShEx schema from the file *dcso-dmp.3.0.2.shex*;	5. On GitHub, copy the content of the DCSO ShEx schema from the file *dcso-dmp.3.0.2.shex*;
6. Paste that schema on the second text area of the RDFShape validation service, (e.g., ShEx);	6. Paste that schema on the left text area of ShEx2 Validator;
7. Define what entity should be validated by adding the text *ex:dmp_1@< dmp>* to the third text area in the validator, (e.g., ShapeMap);	7. Define what entity should be validated by adding the text *< https://w3id.org/dcso /id/example/dmp_1>@< dmp>* to the “Query Map” text area in the validator;
8. Run the validation by clicking the button *Validate*;	8. Run the validation by clicking the button *Validate*;
9. You should get a passing validation.	9. You should get a passing validation.

^a^https://github.com/RDA-DMP-Common/RDA-DMP-Common-Standard/tree/master/ontologies/validation. Accessed 11 Apr 2022

^b^https://github.com/RDA-DMP-Common/RDA-DMP-Common-Standard/tree/master/ontologies/validation. Accessed 11 Apr 2022

^c^https://rdfshape.weso.es/shExValidate. Accessed 09 Mar 2022

^d^http://shex.io/webapps/shex.js/doc/shex-simple.html. Accessed 11 Apr 2022

#### Third stage - human readability and dissemination

By the third stage of the development process most of the functional requirements of DCSO had already been met. The objective was therefore to finalise the development process by addressing requirements that would facilitate its use and adoption. To that effect three tasks were considered: (1) the creation of human-readable descriptions in DCSO Core as well as DCSX, (2) the definition of a persistent namespace for DCSO, and (3) revising and expanding the existing documentation on the various artefacts that comprise DCSO. The creation of human-readable descriptions was done through the usage of *rdfs:comment* descriptions in all created classes, data properties and object properties. The lack of a stable URI to act as DCSO namepsace was one of the issues identified in the initial version of DCSO. To address that issue, and comply with the best practices of using URI namespace as a persistent identifiers, the team resorted to the W3ID^11^ service of the W3C Permanent Identifier Community Group^12^, and registered the identifier ’https://w3id.org/dcso’. Finally, the existing documentation, that consisted mainly of markup files, was revised and deposited, along with DCSO, in a GitHub repository.

### Towards DCSO adoption by the DCS working group

Upon the completion of the first stable version of DCSO (version 3.0.2), it was necessary to have it adopted by the DCS working group as the official serialisation of the DCS application profile. There were however a number of pre-requisites that needed to be met before any proposal for adoption could be formalised. This resulted in DCSO version 4.0.0, which is the latest version in existence. The pre-requisites were as follows:

**Integration of DCSX namespaces into the Validation layer.**

The use of DCSX as an extension of DCSO was rooted on the necessity of having the means to represent controlled vocabularies that applied to a specific set of DCS application profile terms (see “Second stage - DCSX and validation layer” section). However, in order to have a closer representation of the DCS application profile, where the terms covered by DCSX are represented with string values, it was decided that DCSX approach should be abandoned in favour of having these terms represented as data properties in DCSO Core. As a result, the controlled vocabularies were represented as constraints in the validation layer, so as to maintain the validation feature idealised in the original vision of DCSO.

**Full coverage of the DCS application profile by DCSO.**

A fundamental pre-requisite was to ensure that all of the DCS application profile terms were covered by a matching term in DCSO. The methodology adopted to comply with this pre-requisite was to perform a direct comparative analysis between the DCS application profile terms and the first stable version of DCSO (see “First stable version” section). As a result of this analysis, multiple discrepancies were found as it can be seen in Table 2. These discrepancies fell under two categories: Missing terms These DCS application profile terms were found not to have a corresponding match in DCSO. The most common case was the lack of representation for the various *identifier* terms that are associated with multiple DCS application profile concepts. In the first stable version of DCSO, there was an explicit attempt at defining a representation for the DCS application profile identifier concepts, through the *dcso:Id* class, its subclasses (e.g., *dcso:ContactId*, *dcso:ContributorId*, etc.), and the *dcso:identifierType* data property that had the *dcso:Id* class as its domain. However, there was no data property to represent the *identifier* term. The solution to this issue was the creation of the *dcso:identifier* data property, which had as domain the *dcso:Id* class. Three other additional data properties were also created, namely the *dsco:created*, *dcso:modified*, and *dcso:type*. Terms represented with the wrong type These discrepancies were a direct result of the decision to abandon DCSX as an approach to represent DCS application profile terms that specified a set of controlled vocabularies. The solution to this discrepancy was to change the representation type of DCSO terms from object property to data property. As a result the *dcso:hasLanguage*, *dcso:hasGeoLocation*, and *dcso:hasCurrencyCode* DCSO object properties were replaced by the *dcso:language*, *dcso:geoLocation*, and *dcso:currencyCode* DSCO data properties.

**Table 2:** Discrepancies found as a result of the comparative analysis between the DCS application profile and the first stable version of DCSO

DCS Application Profile		DCSO Core	
Concept	Term	Discrepancy	Type
dmp	created	Missing term	Data Property
	language	Wrong type (dcso:hasLanguage)	Object Property
	modified	Missing term	Data Property
dmp_id	identifier	Missing term	Data Property
funder_id	identifier	Missing term	Data Property
grant_id	identifier	Missing term	Data Property
host	geo_location	Wrong type (dcso:hasGeoLocation)	Object Property
metadata	language	Wrong type (dcso:hasLanguage)	Object Property
metadata_standard_id	identifier	Missing term	Data Property
contact_id	identifier	Missing term	Data Property
contributor_id	identifier	Missing term	Data Property
cost	currency_code	Wrong type (dcso:hasCurrencyCode)	Object Property
dataset	language type	Wrong type (dcso:hasLanguage)	Object Property
		Missing term	Data Property
dataset_id	identifier	Missing term	Data Property

**Adoption of DCS application profile name and versioning scheme.**

The current name and versions of DCSO are based on the development of the ontology, without any explicit connection with the reference DCS application profile. In order to ensure consistent DCS application profile usage across its various serialisations, the adoption of the ’maDMP’ term as the semantic serialisation name, in opposition to DCSO, and the adoption of the DCS application profile version number are under consideration. Such a decision would entail that new versions of the ontology would be released along with any revision of the DCS application profile.

**Mechanisms for transformation between JSON and RDF representations of maDMP.**

Due to the popularity of the JSON serialisation of DCS application profile, it is essential for the adoption of DCSO to provide mechanisms for transforming data between JSON and RDF serialisations. To this end, the availability of JavaScript Object Notation for Linked Data (JSON-LD) context to transform maDMP JSON to RDF serialisation and JSON-LD serialiser to transform maDMP RDF to JSON serialisation are important. We provide an initial approach to execute these functions and provides examples of the transformations^13^.

**Exemplifications of funder profile as ontology extensions.**

One of the main advantage of having RDF serialisation of maDMP is its capability to capture data model extensions without having to change the original DCS application profile. To this end, we plan in the future to provide a set of procedures to define such extension to the DCS application profile, and evaluate it to define funder profiles as ontology extensions.

## Utility and discussion

This section focuses on providing a description of the adoption of the DCSO by the Data Stewardship Wizard (DSW), which is one of the most popular DMP creation tools currently available for public usage. Additionally it also features a “Discussion” section, where the benefits of having a semantic representation of a DMP are highlighted.

### Use case: data stewardship wizard

The DSW^14^ is a tool used for data management planning widely used in the European life-sciences Infrastructure for biological Information (ELIXIR) and beyond [19]. Its versatility has been proven in several customisations such as VODAN-in-a-Box solution as a data-entry tool for case report forms [20] or as a FIP Wizard to allow efficient capturing of FAIR Implementation Profiles [21]. The core idea of DSW is allowing for customisation corresponding to funders’ DMP templates. To accomplish this, DSW has a concept of knowledge models and extensible templates for smart questionnaires, where guidance is done in multiple but natural ways – explanations, advice, options for answering, follow-up questions, references, and integrations with application programming interfaces (APIs) to provide answer suggestions. With such a questionnaire, one can get an actual document by selecting the desired export template, e.g., Horizon 2020 DMP template [22]. The export templates are done in Jinja2 templating language^15^; therefore, it can produce any textual format and transform questionnaire replies with any limitations.

During the RDA maDMP Hackathon 2020, a new export template was developed. First, defining the mapping between the core DSW knowledge model and the DCS application profile JSON schema was needed. Some of the information was not covered by then, and thus new questions were added. Also, several of the questions were adjusted or moved; a task facilitated by the DSW migration mechanism making it possible for users to easily upgrade to a newer version without losing all the stored answers. The Jinja2 template for maDMP in JSON is straightforward. It queries the answers using known universally unique identifiers (UUIDs) for the questions, and creates an object according to the predefined JSON schema. Then it plainly pretty-prints the object as a JSON. An export template in DSW may provide several formats. For example, one can export Horizon 2020 DMP documents in the.pdf,.docx,.html,.tex, or.md file formats. DSW also supports RDF export for maDMPs by using DCSO. There is a Jinja2 template for transforming the same object used for JSON to synthesise an RDF file in the Turtle format. It traverses the object (its fields, arrays, nested objects) and outputs the RDF triples according to DCSO. In the first version, the template produced valid RDF but with the use of blank nodes. It turned out that it causes problems with several other tools after being exported from DSW, i.e., lays obstacles in interoperability. The recent version is free of blank nodes by giving every node a unique identifier.

The identifiers of nodes in RDF are of two types. First, there are those concretely defined by JSON schema (and DCSO), e.g., dcso:DMPId or dcso:FunderId. It is verified if such a user-entered identifier is a URI directly or after some transformation (for instance, we may add https://doi.org/ before a Digital Object Identifier (DOI)). In the case of a valid URI, it is used as an identifier for the corresponding node. Then, if it is not a valid URI or the entity does not have an ID defined by DCSO, it still needs a URI. It is synthesised using related URI and question/reply Universally Unique Identifier (UUID), e.g., the URI of the DMP, the URI of the questionnaire and the UUID of dataset reply item. It is also important to point out the integrations in DSW. A user can, for example, select a funder using integration with CrossRef^16^, similarly a license from Wikidata^17^ or affiliation from the Research Organization Registry (ROR)^18^. The URI of an entry is then saved as part of the reply and used in the template corresponding to RDF triples, following the linked data principles.

Finally, Turtle is not the only RDF export format that DSW supports for maDMPs and DCSO. Using rdflib^19^ for automatic transformations allows for export in different formats such as RDF/XML, TRiG, N3, or JSON-LD. The export template is developed as open-source^20^, and anyone can easily contribute to or use it. This approach allows both simple versioning of the template (e.g., when a new version of DCSO is released) and adopting various future extensions to DCSO in independent branches.

### Discussion

The creation of a semantic-based serialisation of the DCS application profile had been identified as a research opportunity by the DCS working group, however it was only after the RDA Hackathon on Machine-Actionable Data Management Plans (see “First stable version” section) that a group of researchers with the adequate expertise to tackle this opportunity was assembled. Having a semantic-based serialisation had the overall goal of facilitating information exchange between different services involved in data management as it becomes independent of their technical implementation. One of the other factors motivating the selection of ontologies was their extensibility, as concepts can be matched or relations established between ontologies covering distinct domains. This is manifested in the DCSO, which makes use of existing domain ontologies such as the World Wide Web Consortium (W3C) DCAT Specification, Dublin Core Metadata Initiative (DCMI) Metadata Terms, and Friend of a Friend (FOAF) to further standardise its content, and thus increase its FAIRness. Additionally, its use of controlled vocabularies (for languages, countries and currencies) further aids in the standardisation of the represented content. All of these factors contribute for the DMP content to be more easily linked to other graphs such as Persistent Identifier (PID) graphs or Research Object graphs. Ontologies also enable reasoning, and, as a consequence, knowledge inference from information explicitly represented [10], which are characteristics that could be explored in the future.

The creation of the DCSO, and more specifically its latest version, is a further step to equip the community with increasingly better solutions for research data management. The creation process of the DCSO was not always ideal, but with each iteration we attempted to address identified challenges and abide by best practices in a continuous cycle of improvement. This continuous cycle of improvement is validated by the adoption of the DSCO by an established DMP creation tool such as the DSW. With DSW now having another means to represent DMP documents, its community is now able find novel ways to exploit this serialisation. As for the DCSO, it will be returned to the community from which it originated (i.e., the DCS working group), in a process that has already began. The DCS working group will face an organisational challenge, as all standards, recommendations, models, or in this case ontologies, require maintenance. The next steps will focus on establishing how this serialisation will be maintained, and by whom. This process will undoubtedly require multiple rounds of revisions and creation of the corresponding documentation. This, however, falls out of the scope of this paper.

## Conclusions

This paper reports on the DCSO creation process up to its latest version (4.0.0), and its path towards adoption by the DCS working group as a serialisation of the DCS application profile. Additionally it also reports on the DCSO’s adoption by the by the DSW, one of most the popular DMP creation tools. DCSO is a semantic representation of the DCS application profile, covering all terms from the DCS application profile whilst also reusing terms from established domain ontologies. Furthermore, DCSO is equipped with a validation layer, which consist of a set of ShEx constraints that allows DMPs represented through DCSO to be validated against the DCS application profile. The DCSO adds to existing DCS application profile serialisations in the sense that it goes beyond just being a representation of data, it allows knowledge to be inferred from data. As such, the DCSO has the potential to expand the use cases for the DCS application profile. However, the DCSO suffers with the limitations associated with formal semantic-based representations. Namely a high cost of entry for both usage and extension, as well as added complexity when compared with other DCS application profile serialisations. These are factors that might challenge the adoption of the DCSO by the community.

The team in charge of the development and maintenance of DCSO strives to follow the best practices in ontology engineering, whilst continuously trying to update and upgrade DCSO and its components. Currently, the team is focusing on preparing a formal proposal for the adoption of DCSO by the DCS working group as an official serialisation of the DCS application profile. This will entail defining how the DCSO will be maintained in the future, which will require a close collaboration with the DCS working group. Additionally, the team is also tackling or planning to tackle the following issues: (1) continued pursue of the integration of terms from other established ontologies into DCSO (e.g., the Data Integration for Grant Ontology (DINGO)^21^), thus enriching the DCS application profile and potentially includes controlled vocabularies associated to all of the relevant concepts; (2) performing semantic validation of DMP documents represented using DCSO, an ambitious but useful feature, in particular for any funding agency stakeholders. Services aiming to perform the task through the usage of DCSO are currently being considered for the creation of proof of concept tools.

## Data Availability

The datasets generated and/or analysed during the current study are available in the DMP Common Standards working group GitHub repository repository, https://github.com/RDA-DMP-Common/RDA-DMP-Common-Standard/tree/master/ontologies

## Abbreviations

API: Application Programming Interface
DC: Dublin Core
DCAT: Data Catalog Vocabulary
DCMI: Dublin Core Metadata Initiative
DCS: DMP Common Standard
DCSO: DMP Common Standard Ontology
DCSX: DCSO Extensions
DINGO: Data Integration for Grant Ontology
DMP: Data Management Plans
DSW: Data Stewardship Wizard
DOI: Digital Object Identifier
ELIXIR: European life-sciences Infrastructure for biological Information
FAIR: Findable, Accessible, Interoperable and Reusable
FOAF: Friend of a Friend
ISO: International Organization for Standardization
JSON: JavaScript Object Notation
JSON-LD: JavaScript Object Notation for Linked Data
maDMP: machine-actionable DMP
LOD: Linked Open Data
OWL: Web Ontology Language
PID: Persistent Identifier
RDA: Research Data Alliance
RDF: Resource Description Framework
ROR: Research Organization Registry
SHACL: Shapes Constraint Language
ShEx: Shape Expression
Turtle: Terse RDF Triple Language
URI: Uniform Resource Identifier
UUID: Universally Unique Identifier
W3C: World Wide Web Consortium
XML: Extensible Markup Language

## Footnotes

1 The Research Data Alliance Website. https://www.rd-alliance.org/rda-europe. Accessed 09 Mar 2022.

2 The DCS application profile JSON serialisation, in GitHub. https://github.com/RDA-DMP-Common/RDA-DMP-Common-Standard/tree/master/examples/JSON. Accessed 09 Mar 2022

3 Definition of application profile, according to Dublin Core. https://www.dublincore.org/resources/glossary/application_profile/. Accessed 09 Mar 2022.

4 Data Catalog Vocabulary - Version 2. https://www.w3.org/TR/vocab-dcat-2/. Accessed 09 Mar 2022.

5 The Dublin Core specification. https://www.dublincore.org/specifications/dublin-core/dcmi-terms/. Accessed 09 Mar 2022.

6 The Turtle syntax. https://www.w3.org/TR/turtle/. Accessed 09 Mar 2022.

7 https://www.wikidata.org/. Accessed 11 Apr 2022.

8 https://www.wikidata.org/wiki/Wikidata:WikiProject_Schemas. Accessed 11 Apr 2022.

9 http://shex.io/webapps/shex.js/doc/shex-simple.html. Accessed 11 Apr 2022.

10 https://shex.io/. Accessed 11 Apr 2022.

11 The W3ID service webpage. https://w3id.org/. Accessed 09 Mar 2022.

12 The W3C Permanent Identifier Community Group website. http://www.w3.org/community/perma-id/. Accessed 09 Mar 2022.

13 The Transformation between DMP Common Standard serialisations application, in GitHub. https://github.com/fekaputra/dcso-json. Accessed 09 Mar 2022.

14 The Data Stewardship Wizard website. https://ds-wizard.org. Accessed 09 Mar 2022.

15 The Jinja project website. https://jinja.palletsprojects.com. Accessed 09 Mar 2022.

16 The CrossRef website. https://www.crossref.org. Accessed 09 Mar 2022.

17 The Wikidata website. https://www.wikidata.org. Accessed 09 Mar 2022.

18 The Research Organization Registry Community website. https://ror.org. Accessed 09 Mar 2022.

19 The rdflib specification. https://rdflib.readthedocs.io. Accessed 09 Mar 2022.

20 The maDMP template for DSW, in GitHub. https://github.com/ds-wizard/madmp-template. Accessed 09 Mar 2022.

21 The Data Integration for Grant Ontology specification. https://dcodings.github.io/DINGO. Accessed 09 Mar 2022.
